# The evolutionary and molecular features of the broad‐host‐range plant pathogen *Sclerotinia sclerotiorum*

**DOI:** 10.1111/mpp.13221

**Published:** 2022-04-11

**Authors:** Mark C. Derbyshire, Toby E. Newman, Yuphin Khentry, Akeem Owolabi Taiwo

**Affiliations:** ^1^ Centre for Crop and Disease Management School of Molecular and Life Sciences Curtin University Perth Western Australia Australia

**Keywords:** broad host range, effector, fungal pathogen, host generalism, quantitative disease resistance, small RNAs, white mould

## Abstract

*Sclerotinia sclerotiorum* is a pathogenic fungus that infects hundreds of plant species, including many of the world's most important crops. Key features of *S. sclerotiorum* include its extraordinary host range, preference for dicotyledonous plants, relatively slow evolution, and production of protein effectors that are active in multiple host species. Plant resistance to this pathogen is highly complex, typically involving numerous polymorphisms with infinitesimally small effects, which makes resistance breeding a major challenge. Due to its economic significance, *S. sclerotiorum* has been subjected to a large amount of molecular and evolutionary research. In this updated pathogen profile, we review the evolutionary and molecular features of *S. sclerotiorum* and discuss avenues for future research into this important species.

## Graphical abstract

This article describes key aspects of the biology of *Sclerotinia sclerotiorum* that have been uncovered since the last pathogen profile was published in 2005, highlighting important research gaps that may be addressed in the future.

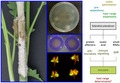

## 1 INTRODUCTION

Some plant pathogens can infect an extremely diverse range of hosts (Newman & Derbyshire, 2020). Among these is the necrotrophic fungus *Sclerotinia sclerotiorum*, which stands out for its ability to cause significant disease in numerous crops. Disease caused by this pathogen is given various names, though one of the most common seems to be sclerotinia stem rot. *S. sclerotiorum* infects crops in almost all agricultural production zones ranging from dry bean (*Phaseolus vulgaris*) in the tropics (Lehner et al., 2017) to canola (*Brassica napus*) in the Mediterranean and temperate climates of Australia, Europe, and North America (Denton‐Giles et al., 2018; Derbyshire & Denton‐Giles, 2016).

Due to its major impact on global agriculture, *S. sclerotiorum* has been the subject of a substantial amount of molecular research spanning several decades. Its ability to infect the model plants *Nicotiana benthamiana* and *Arabidopsis thaliana* makes it an attractive model for research into host generalism. In this review, we highlight recent literature on the genetics and evolutionary and molecular biology of *S. sclerotiorum* and discuss potential avenues for further research.

## 2 THE EXTRAORDINARILY BROAD HOST‐RANGE OF *S. SCLEROTIORUM*

An early study identified 408 plant species in 75 families that are *S. sclerotiorum* hosts (Boland & Hall, 1994). We used a combination of the search terms “*Sclerotinia sclerotiorum*” and “first report” in the Clarivate Analytics Web of Knowledge database (available at the time of writing at apps.webofknowledge.com) and Google to identify reports of *S. sclerotiorum* on new hosts or in new geographical regions since the publication of Boland and Hall. We only included peer‐reviewed publications in academic journals and made note of whether fungal strains were identified as *S. sclerotiorum* using molecular markers (Table S1).

From this survey, we found 48 additional host species in 22 families, five of which are also new. Among the new species identified, 33 were described as hosts using molecular markers to confirm the presence of *S. sclerotiorum*. Most studies used the internal transcribed spacer (ITS) region, though two also used the β‐tubulin gene sequence. The five new families were all identified based on sequencing of the *S. sclerotiorum* ITS region.

Other than dicots, Boland and Hall included 29 monocots, four gymnosperms, and one pteridophyte. Among the additional species we found, there was one monocot and 47 dicots.

Because the vast majority of *S. sclerotiorum* hosts described so far are dicots and *S. sclerotiorum* is not a known pathogen of any economically significant nondicots, we revisited the references used for identification of nondicot parasitism in Boland and Hall (Table S2). We noticed that several nondicot hosts in Boland and Hall were included based on possible misidentifications or colonization of dead tissue in vitro. For example, oat (*Avena* sp.) was included as a host based on reference to growth of sclerotia on plates containing oatmeal agar. Also, *Philodendron selloum* was included as a host based on colonization by *Sclerotium rolfsii*. There were also several references with titles that suggested misidentification but that could not be assessed because we could not source them online. For example, reference 97 (Hysek et al., 1986) is entitled “Mycoflora on stored cereal seed” and reference 144 (Miclauş et al., 1988) is entitled “Presence and spread of *Sclerotinia sclerotiorum* (Lib.) de Bary on seeds of various cereal and industrial crops”, which are both suggestive of postharvest growth on monocot seed rather than infection *sensu stricto*. This is particularly likely given the fact that reference 144 was used to support *S. sclerotiorum* infection of the two major crops wheat (*Triticum aestivum*) and barley (*Hordeum vulgare*), which are both nonhosts of *S. sclerotiorum* (Tian et al., 2020). We identified several further instances where a host was listed but the reference used to support it did not mention it as a host of *S. sclerotiorum*.

Apart from these examples of misidentification, we also found that almost all the references describing nondicot hosts in Boland and Hall were to potentially less reliable literature. For example, references 11, 14, 47, 189, 201, 221, and 235 are plant disease indices published by local government agricultural departments between 1960 and 1984, which did not describe the criteria used to identify plants as *S. sclerotiorum* hosts. In Boland and Hall, these indices were used to designate 21 monocots as hosts of *S. sclerotiorum*. Furthermore, reference 3 (Adams, 1974) is a personal communication within a PhD thesis that is used to identify four monocot species as *S. sclerotiorum* hosts. In most cases, we found no additional support in the academic literature for infection of these nondicot hosts by *S. sclerotiorum*.

The only convincing nondicot hosts we found for *S. sclerotiorum* were Chinese chive (*Allium tuberosum*), which was among the 48 new hosts we identified from our literature survey (Choi et al., 2017), and *Trillium foetidissimum*, which was listed in Boland and Hall. *A. tuberosum* was identified as a host based on the ITS sequence of a fungal strain isolated from it. Furthermore, *A. tuberosum* plants were reinfected with the same strain, confirming pathogenicity. Though no molecular markers were used to identify *T. foetidissimum* as a host, a fungal specimen matching the description of *S. sclerotiorum* was recovered from this species and used to reinfect it, confirming its pathogenicity (Holcomb, 1990).

Because *A. tuberosum* is a host of *S. sclerotiorum*, it seems likely that the other *Allium* species listed in Boland and Hall, *A. sativa* and *A. cepa*, are also hosts of *S. sclerotiorum*. However, references used to support inclusion of these species were not peer reviewed and we could not find additional support for their colonization by *S. sclerotiorum* in the literature. Furthermore, a recent study suggested that the monocot species *Setaria italica* can be parasitized by *S. sclerotiorum*, albeit to a much lesser degree than fully susceptible dicots (Grabowski & Malvick, 2019). This offers some support to inclusion of the species *Setaria viridis* in Boland and Hall, although we could not find any further support in the research literature for its status as an *S. sclerotiorum* host.

Excluding the nondicot references we deemed unreliable, our revised estimate stands at 425 species in 74 families that are documented hosts of *S. sclerotiorum*. However, it is likely there are many as‐yet undocumented hosts. For instance, the United States Department of Agriculture fungal database (available at the time of writing at https://nt.ars‐grin.gov/fungaldatabases/) includes 2048 different host species or varieties that are associated with *S. sclerotiorum* based on various types of literature. It is also possible that reports in Boland and Hall on some nondicot hosts are accurate, although we do not see any convincing evidence for them at this stage. The main findings from this exercise were that (a) despite its broad host range, *S. sclerotiorum* overwhelmingly favours dicotyledonous plants, apart from a few striking exceptions; and (b) there is no support for pathogenesis on the major cereal crops included in Boland and Hall. In this section, we discuss the potential molecular determinants of host range in *S. sclerotiorum* and features of its evolution that might be associated with host generalism.

### 2.1 On the preference of *S. sclerotiorum* for dicotyledonous plants

The vast majority of *S. sclerotiorum* hosts documented by ourselves and Boland and Hall are dicotyledonous. To our knowledge, there have been no direct studies on what limits the host range of *S. sclerotiorum* at the molecular level; however, we can make several speculations.

Numerous species, including *S. sclerotiorum*, produce necrosis‐ and ethylene‐inducing peptide 1‐like proteins (NLPs) (Bashi et al., 2010). These proteins elicit cell death in most dicots but very few monocots (Gijzen & Nürnberger, 2006). NLPs cause necrosis by binding to a specific form of the membrane lipid glycosylinositol phosphorylceramid, widespread among dicots but rare among monocots. One exception is the monocot *Phalaenopsis amabilisi*, which produces both dicot and monocot forms of glycosylinositol phosphorylceramid, making it susceptible to necrosis induced by certain NLPs (Lenarčič et al., 2017). One of the two *S. sclerotiorum* NLP homologues, which both cause necrosis in *N. benthamiana*, is highly expressed during infection and inhibition of its expression is associated with a loss of pathogenicity on *B. napus* (Bashi et al., 2010). However, its necrosis‐inducing activity could not be replicated in a further study (Seifbarghi et al., 2020).

Adding another layer of complexity to the role of NLPs in pathogenesis, they also contain a conserved peptide sequence that elicits an innate immune response in *Arabidopsis* mediated by the leucine‐rich repeat (LRR) receptor‐like protein RLP23 (Albert et al., 2015), and plants that overexpress RLP23 are more resistant to *S. sclerotiorum*. The conservation of NLPs among diverse microbes, including biotrophs, suggests that they may have important functions other than elicitation of necrosis, so they could perhaps behave as elicitors of broad‐spectrum basal immunity (Raaymakers & Van den Ackerveken, 2016). Without knocking out the two *S. sclerotiorum* NLPs, it is not possible to determine whether their primary roles are physiological or associated only with their ability to induce necrosis in dicots; this may be a fruitful area for future research.

Another molecular determinant of the preference of *S. sclerotiorum* for dicot hosts could be oxalic acid. As discussed later, this compound is important for *S. sclerotiorum* pathogenicity on many hosts. Numerous monocot species produce proteins called germins in response to pathogen attack (Davidson et al., 2009). A subclass of these exhibit oxalate oxidase activity and, when expressed in several dicots, can reduce susceptibility to *S. sclerotiorum* by metabolizing oxalic acid (Yang et al., 2019). Perhaps part of the reason *S. sclerotiorum* is unable to parasitize most monocots is their innate ability to metabolize one of its main virulence determinants. To address this question, knocking out monocot oxalate oxidases followed by challenge with *S. sclerotiorum* was suggested by Williams et al. (2011); however, to our knowledge such a study has not been performed.

Although *S. sclerotiorum* does not seem to be a parasite of most monocots, a recent study suggests that it can live endophytically in several of them (Tian et al., 2020). When inoculated onto wheat seedling roots, *S. sclerotiorum* was able to grow inter‐ and intracellularly without causing any visible symptoms. Endophytic growth of *S. sclerotiorum* in wheat led to changes in gene expression reminiscent of the plant immune response to biotrophs. Jasmonic and abscisic acid‐related genes were down‐regulated, whereas salicylic acid‐related genes were up‐regulated. Endophytic growth of *S. sclerotiorum* within monocots also enhanced resistance to several agriculturally relevant diseases, including wheat stripe rust and rice blast. This kind of alternative lifestyle was only previously documented in several species of *Colletotrichum* (Redman et al., 2001). However, because this is only a recently appreciated phenomenon, it may well be common among phytopathogenic fungi. The fact that *S. sclerotiorum* was able to breach the cell wall of the monocots tested without causing necrosis suggests that the difference between *S. sclerotiorum* hosts and nonhosts is related to their cell death responses rather than any physical barriers they can put in place. Perhaps one or more responses to *S. sclerotiorum* triggers of programmed cell death are conserved among dicots but absent in most monocots. This may also be an interesting avenue for further investigation.

### 2.2 The evolutionary features of host generalism

*S. sclerotiorum* is a member of the Sclerotiniaceae clade. The genus *Sclerotinia* is polyphyletic, although the clade containing other well‐known *Sclerotinia* species probably emerged approximately 15 million years ago (Navaud et al., 2018). The best characterized *Sclerotinia* species in this clade other than *S. sclerotiorum* are *S. trifoliorum*, *S. minor*, and *S. nivalis*.

All three of these species have large host and geographic ranges. For example, *S. trifoliorum* hosts include diverse Fabaceae species such as fenugreek (*Trigonella foenum‐graecum*) in Tunisia (Gargouri et al., 2017) and clover (*Trifolium* spp.) and chickpea (*Cicer arietinum*) in the USA (Baturo‐Ciesniewska et al., 2017; Njambere et al., 2014); hosts of *S. minor* include the Asteraceae species lettuce (*Lactuca sativa*) in the USA (Subbarao, 1998) and safflower (*Carthamus tinctorius*) in Turkey (Erper et al., 2020), and the Fabaceae species peanut (*Arachis hypogaea*) in the USA (Crutcher et al., 2018). The most poorly characterized species of the three, *S. nivalis*, also has a broad host range as it can infect plants in at least 10 different families (Wang et al., 2021).

We identified 120, 371, and 13 research papers on *S. trifoliorum*, *S. minor*, and *S. nivalis*, respectively, which in all cases is far fewer than the 4297 for *S. sclerotiorum*. Most research on these species is focused on practical means of control and there has, unfortunately, been little to no molecular research on them to date. Given that these pathogens infect diverse host species but with differing host range restrictions, for example, *S. trifoliorum* is specific to the Fabaceae (Kusch et al., 2022), it would be interesting to compare the molecular mechanisms of their pathogenesis with those of *S. sclerotiorum*.

Some studies have also suggested that different *Sclerotinia* species predominate in different localities in the same geographic region. For instance, Wu and Subbarao (2006) showed that *S. sclerotiorum* was the most prevalent species in lettuce fields in the San Joaquin Valley of California, USA, whereas *S. minor* was the most prevalent species in the Salinas Valley. This was possibly due to poorer survival of the smaller sclerotia of *S. minor* in the conditions of the San Joaquin Valley and a lack of conditions conducive to carpogenic germination of *S. sclerotiorum* in the Salinas Valley.

Despite predominance of different *Sclerotinia* species in different field sites, *S. sclerotiorum* and *S. minor* are frequently found in the same environments (Deng et al., 2021; Marinelli et al., 1998), and Wu and Subbarao (2006) also demonstrated an equal prevalence of *S. sclerotiorum* and *S. minor* in the Santa Maria Valley, suggesting conditions there were equally conducive to growth of both species. The extent to which other *Sclerotinia* species coinhabit similar environmental and host niches, and how this relates to their distribution prior to association with global agriculture, is not known. This may be a good area for future research.

A good starting place for this kind of research would be sequencing and comparison of the complete genomes of these species, which could determine which genes are shared and unique and whether this correlates with host preference or other lifestyle attributes, although only one study so far has used this approach. Kusch et al. (2022) showed that 67.2% of the *S. trifoliorum* genome was syntenic with that of *S. sclerotiorum*. Out of the 10,626 genes predicted in the *S*. *trifoliorum* genome, 93% had homologues in *S. sclerotiorum*. Although the significance of these differences was not the focus of this paper, a more comprehensive view of the differences in gene content across *Sclerotinia*, as was presented for *Botrytis* (Valero‐Jiménez et al., 2019), could inform further research into its evolution.

The best‐characterized Sclerotiniaceae species other than *S. sclerotiorum* is the pathogen *Botrytis cinerea*. Like *S. sclerotiorum*, this species is an extreme host generalist, infecting at least several hundred plant species (Hua et al., 2018). *B. cinerea* probably diverged from *S. sclerotiorum* approximately 40 million years ago (Navaud et al., 2018), so it is likely to exhibit many evolutionary distinctions. With the first available genome sequences of *B. cinerea* and *S. sclerotiorum*, a direct genomic comparison was made (Amselem et al., 2011). On average, protein sequences were 83% identical, indicating a substantial degree of amino acid divergence. Furthermore, there is an expansion in number and diversity of secondary metabolite genes in *B. cinerea*, although the relevance of this to its ecological niche is not known.

Despite the evolutionary distance between *S. sclerotiorum* and *B. cinerea*, there are several examples of virulence mechanisms that are shared between the two. For example, oxalic acid (reviewed in a later section) also contributes to virulence in *B. cinerea* (Rascle et al., 2018). Much like in *S. sclerotiorum*, this may be linked to a general requirement for pH modulation in hosts that have naturally more alkaline tissues (Müller et al., 2018). A recent study on *S. minor* also describes a role for oxalic acid in pathogenicity (Livingstone et al., 2005), suggesting this may be a conserved virulence mechanism in the Sclerotiniaceae.

Infection of many host species is a form of niche generalism. There have not been many studies of the evolution of niche generalism in fungi. However, the few studies that exist on *S. sclerotiorum* and its allies offer some insights into its overall evolutionary dynamics.

The first question that should be answered is how long has the *S. sclerotiorum* lineage been host generalist? The Sclerotiniaceae family, in which *S. sclerotiorum* is placed taxonomically, contains many members that infect a fairly large range of hosts. A joint phylogenetic analysis of the Sclerotiniaceae and 263 of their hosts suggests that the Fabaceae is the most likely ancestral host family of the Sclerotiniaceae (Navaud et al., 2018). About half of the Sclerotiniaceae species included in this study infected plant species that diverged before the appearance of the Fabaceae, suggesting numerous host jumps. One of these host jumps was to the Ranunculales, and it probably occurred with the origin of the *Sclerotinia* genus 20 million years ago. Since then, *S. sclerotiorum* has acquired its several hundred known hosts.

The evolutionary trajectory of the Sclerotiniaceae was also characterized by Navaud et al. (2018), who found that different clades in the Sclerotiniaceae underwent different diversification rates in their evolutionary histories. Early diverging clades, encompassing the genera *Monilinia*, *Encoelia*, and *Ciboria*, had relatively low diversification rates. Intermediate diversification rates were found among species in the more recently diverged genera *Sclerotinia* and *Myriosclerotinia*. The most recently diverged genus, *Botrytis*, exhibited high diversification rates. The intermediate diversification rate observed among *Sclerotinia* and *Myriosclerotinia* lineages had a significant association with host generalism. Development of host generalism was overwhelmingly underpinned by accumulation of new hosts rather than other mechanisms, such as continuing association with a diversifying host lineage.

Host range expansion, such as that observed in *Sclerotinia*, has been well studied in herbivorous insects (Peccoud et al., 2008) though less so in fungi. What has allowed *S. sclerotiorum* to expand its host range to such a degree is still mostly an open research question. Host range expansion may result from development of a preadaptation to the new host followed by colonization and further selection. Conservation of eight important virulence factors suggests the pre‐existence of several of the molecular features required for colonization of contemporary plant hosts in the common ancestor of the Sclerotiniaceae. Positive selection among five of these genes suggests an evolutionary arms race with hosts, consistent with selection following host shifts and range expansions. Interestingly, expression of oxaloacetate acetylhydrolase, which is essential for oxalic acid production (discussed in a later section), was expressed 10 to 300 times higher in host generalists than in specialists, concomitant with accumulation of a larger amount of oxalic acid during infection. This gene could therefore represent a preadaptation that has since increased in expression to facilitate host generalism (Andrew et al., 2012). Future research could focus on the extent of preadaptation and how this has influenced host acquisition and host range limit in *S. sclerotiorum*.

The way that host generalism shapes the genomes of living organisms is not well understood. Host generalist fungi tend to encode longer secreted proteins than host specialists. This is because host generalists must secrete diverse carbohydrate active enzymes to metabolize diverse host substrates. Because mRNA translation is energetically costly and the energy that goes into production of secreted proteins is lost from the cell, translation efficiency is under strong selective pressure in host generalists. In natural populations of *S. sclerotiorum* there is a strong selective constraint on polymorphisms in codons that match the most abundant tRNAs, aligning it with the general model (Badet et al., 2017a).

Additionally, gene length and translation efficiency increase with gene age (Prat et al., 2009), which suggests that host generalists have a slower rate of gene diversification than specialists. This is consistent with weaker selective pressure from hosts, such as that exerted by quantitative disease resistance (discussed in the next section) as opposed to qualitative resistance mediated by resistance genes or all‐or‐nothing susceptibility loci.

## 3 THE MOLECULAR BASIS OF PLANT DEFENCE AGAINST *S. SCLEROTIORUM*

The degree of susceptibility to *S. sclerotiorum* in host populations often varies considerably. However, to our knowledge, complete resistance to *S. sclerotiorum* does not exist in any host species. Continuous variation in disease susceptibility is referred to as quantitative disease resistance and can be controlled by many loci of small effect or fewer loci of relatively large effect (French et al., 2016). In this section we discuss the genetic architecture of *S. sclerotiorum* resistance in host populations and what is known about its genetic control.

### 3.1 Insights into quantitative disease resistance derived from genome‐wide association and quantitative trait locus mapping

We found 29 studies published since 2005, when the last Pathogen Profile was published, reporting associations between molecular markers and resistance to *S. sclerotiorum* in seven host species (Table 1). The proportion of variance (*R* ^2^) explained by the strongest quantitative trait locus (QTL) in each study ranged from 0.08% to 64.4%, averaging 22.65%. Many of the strongest associations were identified in populations with a narrow genetic base. For example, the QTL explaining 64.4% of the variance was identified in a population derived from a cross of two *Pisum sativum* lines (Ashtari Mahini et al., 2020). The second strongest QTL, which explained 48.64% of genetic variation, was also identified in a narrow gene pool developed through interspecific hybridization and backcrossing (Rana et al., 2019). The two strongest associations from wider gene pools were identified in *Glycine max* (soybean), and they explained 32% and 37% of phenotypic variance (Boudhrioua et al., 2020; Moellers et al., 2017).

**TABLE 1:** Analyses of quantitative trait loci involved in resistance to *Sclerotinia sclerotiorum*

Reference	Host	GWAS/biparental mapping/both	Maximum *R* ^2^	Maximum population size	Genes identified experimentally
Talukder et al. (2021)	*Helianthus argophyllus* × *Helianthus annuus* introgression lines	Biparental mapping	22.6	134	None
Ashtari Mahini et al. (2020)	*Pisum sativum*	Biparental mapping	64.3	324	None
Boudhrioua et al. (2020)	*Glycine max*	GWAS	32	127	None
Campa et al. (2020)	*Phaseolus vulgaris*	GWAS	Not discussed	294	None
Rana et al. (2019)	*Brassica juncea* × *Erucastrum cardaminoides* introgression lines	GWAS narrow genetic base	48.64	96	None
Atri et al. (2019)	*Brassica fruticulosa* × *Brassica juncea* introgression lines	GWAS narrow genetic base	16.04	88	None
Wu et al. (2019)	*Brassica napus*	Biparental mapping	22.19	150	None
Badet et al. (2017b)	*Arabidopsis thaliana*	GWAS	Not discussed	84	AT1G20380: prolyl‐oligopeptidase associated with quantitative disease resistance (POQR)
Rana et al. (2017)	*Brassica fruticulosa* × *Brassica juncea* introgression lines	GWAS narrow genetic base	15.28	93	None
Zubrzycki et al. (2017)	*Helianthus annuus*	Biparental mapping	23.87	114	None
Wei et al. (2017)	*Glycine max*	GWAS	4.41	275	None
Talukder et al. (2016)	*Helianthus annuus*	Biparental mapping	31.6	106	None
Gyawali et al. (2016)	*Brassica napus*	GWAS	25	152	None
Wu et al. (2016)	*Brassica napus*	GWAS	6.14	448	None
Amouzadeh et al. (2013)	*Helianthus annuus*	Biparental mapping	3.16	99	None
Wu et al. (2013)	*Brassica napus*	Biparental mapping	32.61	190	Indole glucosinolate methyltransferase (BnaC.IGMT5)
Davar et al. (2010)	*Helianthus annuus*	Biparental mapping	0.08	116	None
Pérez‐Vega et al. (2012)	*Phaseolus vulgaris*	Biparental mapping	40	104	None
Guo et al. (2008)	*Glycine max*	Biparental mapping	15.7	94 lines/45 families	None
Vuong et al. (2008)	*Glycine max*	Biparental mapping	12.1	155	None
Rönicke et al. (2007)	*Helianthus annuus*	Biparental mapping	17.1	283	None
Maxwell et al. (2007)	*Phaseolus vulgaris*	Biparental mapping	20.2	94	None
Zhao et al. (2015)	*Arabidopsis thaliana*	GWAS	22	100	AT4G14147: actin‐related protein complex 4 (ARPC4).
Wei et al. (2016)	*Brassica napus*	GWAS	Not discussed	347	None
Bastien et al. (2014)	*Glycine max*	GWAS	14.5	130	None
Moellers et al. (2017)	*Glycine max*	GWAS	37	466	None
Wen et al. (2018)	*Glycine max*	GWAS	5.2	962	None
Jianan et al. (2021)	*Glycine max*	Both	21.14	261	Glyma.13G072300: glutathione S‐transferase C‐terminal‐like/translation elongation factor EF1B (GmGST).
Yin et al. (2009)	*Brassica napus*	Biparental mapping	36.06	72	None

Abbreviation: GWAS, genome‐wide association study.

It appears then that resistance to *S. sclerotiorum* is complex and controlled by numerous genetic loci of relatively small effect and some loci of slightly larger effect in some host species. Although association mapping can be a useful tool for understanding genetic architecture and identifying regions of interest for further investigation, it does not provide a conclusive understanding of trait genetic control. To understand this, alleles within QTLs must be experimentally analysed to determine their contributions to the trait. Out of 29 association mapping studies, we find that four included follow‐up investigations of QTLs.

Two of these were performed in *Arabidopsis* by the same research group in the last 4 years, which is testament to the utility of this species as a model organism. In the first of these studies, a missense mutation strongly associated with disease resistance was identified in a prolyl‐oligopeptidase (given the name Prolyl‐Oligopeptidase associated with Quantitative Resistance [POQR]). *Arabidopsis* null mutants for this gene and *Solanum lycopersicum* (tomato) virus‐induced gene silencing lines for its homologue were more susceptible to *S. sclerotiorum* (Badet et al., 2017b). The POQR‐mediated resistance mechanism was not addressed in this study. However, the authors speculate that POQR could be involved in maturation of cyclic peptides, many of which have antifungal properties (Tavormina et al., 2015).

The second *Arabidopsis* study focused on a gene involved in actin reorganization during pathogen attack. A promoter insertion in a gene encoding an actin‐related protein complex 4 protein causes increased pathogen‐responsive gene expression in some *Arabidopsis* accessions. This gene has pleiotropic impacts on plant hormone‐mediated defence responses, which are linked with callose deposition at infection sites (Badet et al., 2018).

Two further studies in crop plants have involved follow‐up experiments providing some indication of the causal genes behind QTLs. A *G. max* glutathione *S*‐transferase was found close to a QTL detected in both a biparental population and a diverse germplasm set. Plants overexpressing this gene were more *S. sclerotiorum*‐resistant than the wild type (Jianan et al., 2021). Furthermore, this gene was induced to a greater level under pathogen attack in resistant plants compared with susceptible plants. Glutathione *S*‐transferases have many functions in abiotic stress resistance, such as detoxification of phytotoxins and elimination of reactive oxygen species (Gullner et al., 2018). However, the precise role of this gene in the response to *S. sclerotiorum* was not investigated.

In *B. napus*, an indole glucosinolate methyltransferase was associated with a QTL detected in multiple environments. This gene contained single nucleotide polymorphisms with different alleles in the parents of the biparental population used to identify the QTL, and it was also up‐regulated during infection. No further analyses were performed on this gene, though it is a strong quantitative disease resistance gene candidate (Wu et al., 2013).

Overall, we find that despite the large amount of literature devoted to QTL mapping for *S. sclerotiorum* resistance in diverse hosts, such studies are seldom followed up with experimentation to understand the genetic basis of quantitative disease resistance. Those that do follow up on the initial mapping analysis have barely scratched the surface of the genetic basis of quantitative disease resistance to *S. sclerotiorum*. We consider this a fruitful area for future investigation, which may have important implications for disease management through resistance breeding.

### 3.2 Insights into general host responses to *S. sclerotiorum* derived from molecular studies

Several studies have also shed light on the molecular bases of plant responses to *S. sclerotiorum* using techniques such as RNA sequencing, reverse genetics, and simple allelic comparisons. Many of these are reviewed in Wang et al. (2019b), so we keep this review mostly focused on articles published since 2019.

A common theme among these studies is the involvement of plant basal immunity in response to *S. sclerotiorum*. The receptor‐like kinases BAK1 and SOBIR1 have essential and often overlapping roles in both basal immunity and effector‐triggered immunity (Fradin et al., 2009; Jonge et al., 2012; Liebrand et al., 2014; Sun et al., 2013). These proteins are thought to have central coordinating roles in mounting pathogen defence responses and may have been co‐evolving since the time land plants first appeared (Liebrand et al., 2014).

Both BAK1 and SOBIR1 have roles in resistance to *S. sclerotiorum* in *Arabidopsis* (Zhang et al., 2013). An *S. sclerotiorum* culture filtrate fraction was found to elicit typical microbe‐associated molecular pattern‐triggered immunity responses, such as production of the stress hormone ethylene. The receptor‐like protein RLP30 may be the receptor for the elicitor in this culture filtrate fraction. The fact that *RLP30*, *BAK1*, and *SOBIR1* knockout lines are all hypersusceptible to *S. sclerotiorum* suggests that RLP30 requires both BAK1 and SOBIR1 for its function. Interestingly, several *Arabidopsis* accessions with missense mutations in *RLP30* are hypersusceptible to *S. sclerotiorum*, suggesting aspects of the innate immune response are involved in quantitative *S. sclerotiorum* resistance.

In addition to their role in mounting an immune response to an unidentified elicitor via RLP30, BAK1 and SOBIR1 are essential for the necrotizing activity of five *S. sclerotiorum* effectors (Seifbarghi et al., 2020). This could be a form of effector‐triggered susceptibility, whereby a necrotrophic pathogen subverts the host's hypersensitive response system to trigger cell death (De Wit et al., 2009). One of the six effectors tested did not require a secretion signal for induction of necrosis in *N. benthamiana*, suggesting that it does not act in the apoplast. This was also the only effector that did not require BAK1 and SOBIR1 to elicit necrosis, which is not surprising because these two proteins are hubs for the transduction of signals from the external environment. The roles of these proteins in *S. sclerotiorum* virulence and their receptors are currently not known. However, it is possible that they interact with specific receptor‐like proteins whose presence or absence contributes to quantitative disease resistance in natural populations.

Echoing these findings, a recent study showed that a nucleotide‐binding site (NBS)‐LRR protein is responsible for enhanced susceptibility to *S. sclerotiorum* (Barbacci et al., 2020). Knockout lines missing this NBS‐LRR protein and natural accessions with mutations within it were more resistant to *S. sclerotiorum*, suggesting it has a role in susceptibility. NBS‐LRR proteins typically trigger hypersensitive cell death in response to intracellular pathogen effectors (McHale et al., 2006). If the pathogen is a biotroph, this response reduces its spread. However, if the pathogen is a necrotroph, like *S. sclerotiorum*, the hypersensitive cell death may facilitate colonization. The contribution of NBS‐LRR proteins to quantitative disease susceptibility to host generalist necrotrophs is not well understood, and *S. sclerotiorum* could be a good organism in which to investigate it.

Plants also detect pathogens through G‐proteins and G‐protein‐coupled receptors (Zhang et al., 2012), in particular, the “extra‐large” G‐protein (XLG) subfamily (Urano et al., 2012). Three XLGs in *Brassica juncea* are important for resistance to *S. sclerotiorum*, because independent RNA interference (RNAi) lines for these three genes are all more susceptible to *S. sclerotiorum* than the wild type (Tiwari et al., 2021). However, there is much to be uncovered about how XLGs mediate defence to *S. sclerotiorum* and their contribution to quantitative disease resistance.

Once a pathogen has been detected by plasma membrane receptors, the signal is transduced by mitogen‐activated protein kinases (MAPKs) (Meng & Zhang, 2013). A key MAPK immune signalling pathway in *Arabidopsis* involves the two MAPKs MPK3 and MPK6 (Asai et al., 2002). In *B. napus*, both MPK3 and MPK6 have a role in the response to *S. sclerotiorum*. For both genes, overexpression and RNA silencing cause an increase and decrease in *S. sclerotiorum* resistance, respectively (Wang et al., 2019a, 2020b). Both genes also harbour polymorphisms associated with *S. sclerotiorum* resistance in natural populations, suggesting they may be another aspect of innate immunity involved in quantitative disease resistance. In addition to MPK3 and MPK6, MPK4 also has a role in the *B. napus* response to *S. sclerotiorum* (Wang et al., 2009), though its role in quantitative disease resistance is not known.

Once a pathogen has been recognized and the signal transduced, the plant must mount an immune response. This response is mediated by phytohormones such as salicylic acid, jasmonic acid, abscisic acid, ethylene, and brassinosteroids (Verma et al., 2016). Salicylic acid and jasmonic acid were established initially as mediators of distinct responses to biotrophs and necrotrophs, respectively (Thomma et al., 1998). The two phytohormones also seem to antagonize each other (Van der Does et al., 2013; Niki et al., 1998; Proietti et al., 2013). However, more recent studies outside of *Arabidopsis* suggest that salicylic acid may also be required for resistance to necrotrophs. For example, *S. lycopersicum* plants expressing the bacterial gene *nahG*, which encodes a salicylic acid‐degrading enzyme, are more susceptible to the necrotroph *Alternaria solani* than wild‐type plants (Brouwer et al., 2020).

Plant hormone studies in *Arabidopsis* have produced mixed results regarding the relative contributions of jasmonic acid and salicylic acid to defence against *S. sclerotiorum*. The *Arabidopsis* gene *Nonexpressor of PR genes 1* (*NPR1*) responds to salicylic acid to induce expression of defence genes such as *Pathogenesis‐related 1* (*PR1*) (Cao et al., 1994). It is a central coordinator of the salicylic acid response and plants lacking it are salicylic acid‐insensitive. *Arabidopsis npr1* null mutants were initially shown to be hypersusceptible to *S. sclerotiorum*, suggesting a role for salicylic acid in plant defence against it (Guo & Stotz, 2007). Jasmonic acid‐insensitive null mutants such as *coi1* are also hypersusceptible to *S. sclerotiorum*, which would be expected for a necrotrophic pathogen. However, a later study showed that *npr1* mutant *Arabidopsis* lines maintained wild‐type susceptibility to *S*. *sclerotiorum*, although the hypersusceptibility of *coi1* was reproducible (Perchepied et al., 2010).

Recent studies in *B. napus* have also demonstrated a role for *NPR1* in resistance to *S. sclerotiorum*. *B. napus* RNAi lines expressing a lower level of *NPR1* were more susceptible to *S. sclerotiorum*, despite the observation that *NPR1* was down‐regulated in response to it. *NPR1* RNAi lines expressed jasmonic acid‐responsive genes to a much higher level, suggesting that up‐regulation of the jasmonic acid pathway could not compensate for a reduction in salicylic acid responsiveness (Wang et al., 2020a). A further study showed that overexpression of *NPR1* in *B. napus* increases resistance to *S. sclerotiorum* (Wang et al., 2009). This study also showed that exogenous application of salicylic acid enhanced resistance to *S. sclerotiorum*, supporting the importance of this phytohormone in mounting a defence response to it.

Echoing these studies, the *Arabidopsis* secreted lipase GDSL1 may contribute to defence against *S. sclerotiorum* by increasing salicylic acid and decreasing jasmonic acid production (Ding et al., 2020). *B. napus* lines overexpressing the *Arabidopsis GDSL1* gene exhibited elevated levels of salicylic acid and reduced levels of jasmonic acid, concomitant with induction and repression of salicylic acid‐ and jasmonic acid‐responsive genes, respectively. The genes induced in *GDSL1*‐overexpressing lines included *NPR1* and downstream defence response genes such as *PR1*. The contribution of this lipase gene to defence may be via generation of phosphatidic acid, which is a key signalling molecule associated previously with the reactive oxygen species burst and hormonal induction.

Based on these studies it is clear that salicylic acid is an important mediator of defences to *S. sclerotiorum*. However, it is unclear at which infection stage this defence system is most relevant. Some research suggests that *S. sclerotiorum* spends the first few hours of its lifecycle growing in the apoplast, secreting molecules to dampen host immune responses. This noninvasive growth is maintained at hyphal tips and followed by an expanding necrotic lesion (Kabbage et al., 2015). It is possible that salicylic acid is most important as a presymptomatic first responder to *S. sclerotiorum*, when it is becoming established in the intercellular space. So far, the overall contribution of allelic variation in plant hormone‐associated genes to quantitative *S. sclerotiorum* resistance has not been studied, representing another avenue for future research.

Once plants have transduced the pathogen attack signal, plant defence begins with expression of genes with functions ranging from antimicrobial metabolite production to development of defensive barriers at pathogen entry sites. A recent study suggests resistant *G. max* plants reprogramme the phenylpropanoid pathway in response to *S. sclerotiorum* infection, leading to accumulation of antifungal metabolites such as cinnaminic, benzoic, and ferulic acids. Stem extracts from resistant plants inhibited growth of *S. sclerotiorum* in vitro. Furthermore, *Saccharomyces cerevisiae* knockouts for phospholipid and sterol biosynthesis genes were hypersusceptible to these stem extracts, suggesting a possible mechanism for antifungal activity. Mutants for the ergosterol biosynthesis gene *ERG6* had the strongest sensitivity to the stem extracts, suggesting that ergosterol biosynthesis is a major target of *G. max* antifungal defence compounds, which is analogous to the CYP51‐inhibiting triazole class of fungicides (Ranjan et al., 2019).

Most studies so far consider the responses of individual species to *S. sclerotiorum* in isolation. Perhaps key questions going forward are what are the differences and commonalities between the diverse plant species infected by *S. sclerotiorum*? And how does *S. sclerotiorum* exploit shared features of its hosts whilst circumventing a huge array of different defensive strategies?

## 4 THE PARASITIC TOOLKIT OF *S. SCLEROTIORUM*

*S. sclerotiorum* exhibits a complex array of molecular processes that allow it to infect plants. In this section, we discuss the key molecular components of *S. sclerotiorum* pathogenesis. Since the development of gene knockout methods for *S. sclerotiorum*, many studies have investigated the roles of pleiotropic genes in growth, development, and infection. However, we limit this section to *S. sclerotiorum* molecules with direct roles in host cell manipulation, as we believe these have the most clearly defined impacts on the interaction. For a comprehensive overview of pathogen virulence determinants, we direct readers to a recent review (Xia et al., 2020b).

### 4.1 Oxalic acid

Oxalic acid is an organic acid found in animals, plants, bacteria, and fungi (Dutton & Evans, 1996). There is a long history of research into oxalic acid in *S. sclerotiorum*. This research has been comprehensively reviewed and synthesized into a theory of virulence by Xu et al. (2018). Therefore, we keep this section brief, giving some background and highlighting two of the most recent *S. sclerotiorum* oxalic acid studies.

Much of the early research on *S. sclerotiorum* oxalic acid was conducted on UV‐induced mutants producing negligible amounts of oxalic acid compared to the wild type (Dutton & Evans, 1996; Godoy et al., 1990). Their inability to infect any host species led to the conclusion that oxalic acid is a pathogenicity determinant. This line of enquiry culminated in a landmark study showing that oxalic acid can both dampen host defences early during infection and induce programmed cell death later during infection by manipulating the host cell redox environment (Williams et al., 2011). This theory was based partially on the observation that UV‐induced mutants were not able to dampen the oxidative burst early during infection.

However, more recent studies using defined null mutants for the oxalic acid biosynthesis gene *oxaloacetate acetylhydrolase* (*oah*) have produced conflicting results. The first of these studies found that, as well as not producing oxalic acid, *oah* null mutants formed aberrant sclerotia and could not form compound appressoria (Liang et al., 2015). Although oxalic acid production was restored in complementation strains, these defects were not, so they were probably not linked to a lack of oxalic acid. To test the effects of oxalic acid on pathogenicity, Liang et al. infected wounded plants to allow for penetration of host tissues without appressoria. This showed that both the UV‐induced mutant strains and *oah* null mutants produced limited lesions in planta. The *oah* null mutants were similar to the UV‐induced mutants in this regard but produced slightly larger lesions possibly because of their higher growth rate.

A further study developed independent *oah* null strains, which also could not produce oxalic acid. Despite their lack of oxalic acid, these strains produced the weaker fumaric acid, which could lower the pH of the medium but to a lesser extent than oxalic acid. Under alkaline conditions, growth was reduced and sclerotia were not properly formed, but this was because the *oah* null mutants could not reduce the pH of the medium as much as the wild type. These strains produced disease lesions similar to those produced by the wild type on faba bean. However, pathogenicity was reduced by approximately 30%–40% on pea, severely restricted on green bean, and almost abolished on soybean. Defects on green bean were rescued by artificial lowering of the pH but not with addition of potassium oxalate. Furthermore, the sizes of lesions formed by *oah* null mutants were dependent on the ambient pH of host tissue and host buffering capacity. All the defects observed in these strains were rescued by complementation, suggesting no genetic abnormalities other than disruption of *oah* (Xu et al., 2015). A further study has independently shown similar phenotypes in *oah* null mutants in different strains using CRISPR/Cas9 insertional mutagenesis (Jingtao et al., 2018).

These data suggest that the main contribution of oxalic acid to the virulence of *S. sclerotiorum* is reduction of the pH of host tissues, rather than more specific effects on host physiology. These studies also suggest that the oxalic acid UV‐induced mutants harbour unknown defects that inhibit their normal development. Interestingly, these strains are able to produce some oxalic acid in response to ambient pH but their growth rates do not change and they do not produce sclerotia even when the pH of the medium is lowered artificially (Godoy et al., 1990; Liang et al., 2015; Xu et al., 2018). This suggests that they are unable to regulate expression of development‐ and growth‐related genes that would normally respond to a low pH. To our knowledge, there have been no additional studies of the role of oxalic acid in the virulence of *S. sclerotiorum*. Future research could be directed not only toward deepening our understanding of this elusive compound, but also toward identification of the main genetic determinants of the UV‐induced mutant phenotypes.

### 4.2 Proteinaceous effectors

During infection, plant pathogens secrete small proteins known as effectors, which promote disease (Toruño et al., 2016). In the last decade, several *S. sclerotiorum* putative effectors have been discovered.

The majority of characterized *S. sclerotiorum* putative proteinaceous effectors induce cell death in host tissue. As it is a necrotroph, induction of host cell death is critical for *S. sclerotiorum* to acquire nutrients. The two NLPs SsNEP1 and SsNEP2 were the first *S. sclerotiorum* effectors identified and were shown to induce cell death by *Agrobacterium tumefaciens*‐mediated expression in *N*. *benthamiana* leaves (Bashi et al., 2010). This method has since been used to characterize the additional necrosis‐inducing *S. sclerotiorum* effectors Ss‐Caf1, SsSSVP1, SsCP1, and six “SsNEs” (Lyu et al., 2016; Seifbarghi et al., 2020; Xiao et al., 2014; Yang et al., 2018).

Ss‐Caf1 was identified by transfer DNA insertion mutagenesis. A mutant with a transfer DNA insertion in the *Ss‐Caf1* gene had reduced pathogenicity, produced aberrant sclerotia, and lacked compound appressoria. In addition, expression of *Ss‐Caf1* lacking its native signal peptide in *N. benthamiana* induced cell death, indicating that the mature protein could act as an intracellular necrosis‐inducing effector (Xiao et al., 2014).

Like Ss‐Caf1, SsSSVP1 is another intracellular necrosis‐inducing effector. It physically interacts with host QCR8, a subunit of the cytochrome *b*–*c*_1_ complex of the mitochondrial respiratory chain, perturbing its localization to mitochondria. Silencing of *SsSSVP1* caused a minor but significant growth reduction in vitro and a more pronounced reduction in pathogenicity (Lyu et al., 2016). Thus, Ss‐Caf1 and SsSSVP1 may be both developmental and pathogenicity factors.

Yang et al. (2018) characterized an *S. sclerotiorum* protein of the cerato‐platanin family (SsCP1), which has diverse roles in fungal pathogenicity. SsCP1 is required for virulence and induces cell death in *N. benthamiana* both intra‐ and extracellularly. However, cell death could only be induced by viral‐mediated overexpression using a tobacco rattle virus vector and was not replicated in a later study (Seifbarghi et al., 2020; Yang et al., 2018). SsCP1 may interact with PR1 in the apoplast, suppressing its antifungal activity. However, paradoxically, *SsCP1* overexpression in *Arabidopsis* resulted in elevated levels of salicylic acid and increased resistance to several fungal pathogens, which is surprising given the role of SsCP1 in *S. sclerotiorum* virulence and the fact it may target PR1. Further research is required to elucidate the impact of the SsCP1 interaction with PR1 on the plant immune response during *S. sclerotiorum* infection, specifically whether the immune system is activated or inhibited by SsCP1.

In addition to the necrosis‐inducing effectors, the effector SsITL contributes to pathogenicity by dampening the plant immune response. Upon infection, *SsITL* is up‐regulated at 1.5 and 3 h postinoculation of *Arabidopsis* plants, suggesting it plays a role in early infection stages. SsITL may enter host cells and suppress expression of jasmonic acid/ethylene‐ and salicylic acid‐responsive genes, reducing host immunity (Zhu et al., 2013). Recently, Tang et al. (2020) found that SsITL physically interacts with a chloroplast‐localized calcium‐sensing receptor, inhibiting salicylic acid accumulation. The authors speculate that SsITL also interacts with other host components to perturb the jasmonic acid/ethylene signalling pathway. Apart from its role as an effector, SsITL is also important for normal hyphal growth, sclerotial development, and carpogenic germination (Zhu et al., 2013).

The putative *S. sclerotiorum* effector SsCM1 may also interfere with salicylic acid accumulation (Kabbage et al., 2013). SsCM1 is a predicted chorismate mutase with structural similarity to the *Ustilago maydis* effector Cmu1, which is required for virulence on maize through its role in reducing salicylic acid accumulation (Djamei et al., 2011). Surprisingly, the role of SsCM1 in *S. sclerotiorum* virulence has not been published, so it remains a putative effector.

SsCVNH is an effector up‐regulated during infection. Like the necrosis‐inducing proteins Ss‐Caf1 and SsSSVP1 and the immunity‐suppressing protein SsITL, SsCVNH has roles in growth, sclerotia formation, and virulence on *B. napus* (Lyu et al., 2015). The mechanism of action is unknown; however, the presence of a CVNH carbohydrate‐binding domain leads us to speculate that SsCVNH may bind to fungal cell walls to protect against degradation or bind to chitin oligomers to evade pathogen perception by host extracellular receptors, as described for several LysM domain‐containing fungal effectors (e.g., Sánchez‐Vallet et al., 2020). Finally, a putative secreted effector, ssv263, which is an orthologue of a secreted *B. cinerea* protein, is required for virulence on *B. napus* (Liang et al., 2013), although little more is known about this effector.

Investigation into proteinaceous effectors has revealed that *S. sclerotiorum* interacts with its hosts in a much more sophisticated manner than once thought. Understanding the mechanism by which *S. sclerotiorum* proteinaceous effectors function has the potential to open up novel approaches to improving genetic resistance to *S. sclerotiorum* in crops. For example, Zhang et al. (2021) used CRISPR/Cas9 to edit one or more copies of the SsSSVP1 target QCR8 in *B. napus*. Mutants had improved resistance to *S. sclerotiorum* and *B. cinerea* and, importantly, no significant difference in key agronomic traits. Identification of the host targets of other *S. sclerotiorum* effectors represents a promising strategy to identify host resistance or susceptibility genes. The identified genes or their homologues may be good candidates for gene editing to enhance *S. sclerotiorum* resistance in economically important crops.

### 4.3 Metabolism of host defence compounds

To defend themselves against pathogens and herbivores, plants produce a wide array of constitutive and inducible toxic compounds (Tiku, 2020). Glucosinolates are a well‐known class of compound produced by plants with roles in defence against both herbivores and pathogens (Buxdorf et al., 2013; Rask et al., 2000). These compounds are constitutively produced by plants in the order Brassicales, and in response to tissue damage they are activated by enzymes called myrosinases to produce toxic isothiocyanates and nitriles (Rask et al., 2000). To infect plants in the order Brassicales, pathogens must somehow circumvent this aspect of innate immunity.

*S. sclerotiorum* may achieve this by degrading isothiocyanates using the conserved enzyme SsSaxA. Purified SsSaxA is able to hydrolyse several isothiocyanates in vitro, and *S. sclerotiorum* knockout strains missing the gene encoding it were more sensitive to isothiocyanates. Furthermore, *SsSaxA* knockout strains were less pathogenic on wild‐type plants, suggesting that metabolism of isothiocyanates is important for virulence (Chen et al., 2020).

Transcriptomic studies have also hinted at the importance of metabolism of diverse host antimicrobial metabolites in *S. sclerotiorum* pathogenesis. Allan et al. (2019) identified 53 genes that were differentially expressed during infection of the different host species *Lupinus angustifolius* (lupin) and *B. napus*. Several of these genes contained domains linked with metabolism and efflux of xenobiotic compounds, such as cytochrome P450, major facilitator superfamily, and tannase domains. Echoing these findings, Kusch et al. (2022) showed that colonization of distinct host species led to a specific signature of cytochrome P450 induction. This was in line with a wider observation that infection of different host species induces different transcriptional programmes in *S. sclerotiorum*. Interestingly, a cluster of 70 genes was up‐regulated in *S. sclerotiorum* during both infection of *Arabidopsis* and exposure to the Brassicales‐specific antimicrobial camalexin. These genes were conserved in *S. trifoliorum*, which cannot infect Brassicales species, although they were not induced in this species during exposure to camalexin. Enrichment of a specific promoter motif upstream of *S. sclerotiorum* genes but not upstream of their *S. trifoliorum* homologues suggests acquisition of Brassicales hosts was facilitated by *cis‐*regulatory variation leading to a host family‐specific transcriptional response. This echoes previously mentioned findings by Andrew et al. (2012) and suggests that preadaptation to new host environments through a core gene arsenal has been a major factor in the host range expansion of *S. sclerotiorum*. A key question for future research is whether the antimicrobial metabolites produced by most monocots represent a barrier to *S. sclerotiorum* infection.

## 5 SMALL RNAS: A NEW FRONTIER IN THE MOLECULAR BIOLOGY OF *S. SCLEROTIORUM*?

Research in the last 10 years has opened several new frontiers in molecular plant pathology. One of the foremost among these is the understanding that small RNAs (20–25‐nucleotide noncoding RNAs) can be transported from pathogen to host and vice versa (known as cross‐kingdom RNAi) (Cai et al., 2018; Weiberg et al., 2013). These molecules enter the RNAi pathway, guiding a group of enzymes to complementary mRNAs, which are down‐regulated through degradation or translational inhibition (Weiberg & Jin, 2015). Cross‐kingdom RNAi has been described in the host generalist *B. cinerea*, which is closely related to *S. sclerotiorum*, and the host specialists *Puccinia striiformis* and *Fusarium oxysporum* f. sp. *lycopersici* (Ji et al., 2021; Wang et al., 2017a, 2017b). However, evidence for the phenomenon is strikingly absent in the host specialist *Zymoseptoria tritici* (Kettles et al., 2019; Ma et al., 2020).

Preliminary evidence suggests cross‐kingdom RNAi is a key component of *S. sclerotiorum* pathogenesis. Derbyshire et al. (2019) found that *S. sclerotiorum* expresses several hundred small RNAs during infection of both *P. vulgaris* and *Arabidopsis*. The predicted targets of these small RNAs in *Arabidopsis* were significantly less expressed during infection, possibly indicative of silencing by fungal small RNAs. Furthermore, they contained variants associated with quantitative *S. sclerotiorum* resistance in a natural population. Two of the three *Arabidopsis* knockout lines for predicted *S. sclerotiorum* small RNA target genes tested in this study were more susceptible to infection than the wild type, providing an evolutionary reason for the interaction between pathogen small RNAs and these specific mRNAs. However, this study stopped short of direct evidence for interaction between *S. sclerotiorum* small RNAs and plant mRNAs. The roles of small RNAs in *S. sclerotiorum* pathogenesis have thus only just begun to be explored, and future studies may address many important questions regarding their specific functions.

As well as crossing species barriers, small RNAs may also regulate endogenous gene expression. In *S. sclerotiorum*, endogenous small RNAs may regulate genes controlling sclerotial development (Xia et al., 2020a). Other studies on *S. sclerotiorum* have explored the role of small RNAs in response to viruses (Neupane et al., 2019). However, endogenous RNAi in *S. sclerotiorum* is still poorly understood, and thus represents a good area for future investigation.

The fact that the RNAi pathway is active in *S. sclerotiorum* makes it a potential target of novel disease control methods such as host‐induced and spray‐induced gene silencing. Expression of double‐stranded RNAs targeting the *S. sclerotiorum* oxalic acid biosynthesis gene *oah* led to reduced disease susceptibility in *G. max* (McCaghey et al., 2021). In vitro analyses suggested that these double‐stranded RNAs could be taken up by fungal hyphae, leading to the conclusion that they were probably transported from host to pathogen. Based on our understanding of RNAi in fungi, double‐stranded RNAs such as these would then probably enter the endogenous RNAi pathway, producing small RNAs that regulate target gene expression (Dang et al., 2011). Uptake of double‐stranded RNAs is particularly likely in *S. sclerotiorum*, which also makes it highly amenable to topical application of double‐stranded RNAs targeting virulence genes (Qiao et al., 2021). How *S. sclerotiorum* takes up double‐stranded RNAs and the potential for evolution of resistance to them may be important future research questions.

## 6 CONCLUSIONS

In this review, we summarize recent advancements in our understanding of the evolutionary and molecular biology of *S. sclerotiorum* from the perspectives of both host and pathogen. We highlight several emerging areas for future study such as the molecular basis for preference of dicotyledonous plants, the genetic basis of quantitative disease resistance, and the multifaceted roles of small RNAs in fungal development and pathogenicity. As genomic and RNA sequencing studies accrue and Big Data biology becomes the norm, we anticipate many exciting new *S. sclerotiorum* discoveries in the decades to come.

## CONFLICT OF INTEREST

The authors declare no conflict of interest.

## Supporting information


**TABLE S1** All first reports of *Sclerotinia sclerotiorum* since the publication of Boland and Hall (1994)

**TABLE S2** Investigation of references for nondicot hosts in Boland and Hall and rationale for inclusion in updated host list

## Data Availability

Data sharing is not applicable to this article as no new data were created or analysed.
